# Some Notes on the Brewers' Exhibition

**Published:** 1906-10-27

**Authors:** 


### SOME NOTES ON THE BREWERS' EXHIBITION.

While the annual Brewers' Exhibition is remarkable for its commercial value it is also remarkable for the number and variety of articles that are exhibited there. Our interest centres in the quality of the goods rather than in the quantity. Of the many excellent exhibits special mention, although that a short one, should be made of the following:—

Messrs. R. P. Cully and Company, Limited, agent for Worthington's ales, occupy an old-fashioned, timbered, wainscotted inn of the fifteenth-century period. To descant upon the merits of "Worthington's" would seem almost needless. It is an exceedingly nourishing and appetising article of diet, being prepared solely from specially prepared malt and hops. Of its purity as a beverage it is enough to state that the microscope is freely used in all stages of its preparation, and the firm was the first to systematically use this instrument of research in beer-making and to provide a specially-fitted laboratory for the purpose.

Pure light lager Pilsener is so difficult to obtain in England that it is pleasing to note that Peter Stoeri (38-40 Craven Street, N.) specially import an exceedingly fine Munich Kindlbrew lager beer. It is guaranteed brewed from pure malt and hops only, no chemicals whatever being employed in its manufacture. The name of Allsopp is one to conjure with. The purity and excellence of Allsopp's lager and pale ale is too well known to require restatement here. They were the original brewers of India pale ale, and the pioneers of the lager-beer trade in this country. Their products are manufactured from malt and hops and kept in sterilised bottles. Messrs. Bass, who receive their visitors in a very nice reception-room, need no further mention than the fact that the excellent quality of their goods is still maintained.

Rosbach, Limited, are the only firm exhibiting mineral waters. Their business in a continued state of progression speaks for the quality of the article submitted to the public. A very ingenious invention was displayed by Messrs. Idris and Company. It is termed "a waterway of china," and it is the only syphon-top manufactured that absolutely prevents metallic contact. The aerated water passes up the syphon tube straight through the porcelain spout, and this arrangement permits of the free passage of the water without any valvular obstruction, and thus prevents loss of gas or aerating efficiency. The hygienic advantage of this can be better seen than described, but it will certainly soon be appreciated by the general public and as a consequence increased sales of Idris may well be looked for.

Messrs. Anderson's Red Star and White Star whiskies are excellent liqueur whiskies. They are refined and elegant blends noted for their delicacy of flavour and excellence of quality. The "Three Star" is a particularly high-grade spirit, thoroughly matured by long storage in sherry wood, and is indeed a good old liqueur brand.

Messrs. MacLachlan's Three Star Iona needs little recommendation. It is smooth to the palate, and possesses the chief desiderata of whisky—purity, fine quality, and undoubted maturity.

Messrs. W. M. Still and Company, 24 Charles Street, Hatton Garden, E.C., were showing Jones and Still's patent boiling water apparatus with pillar fount which should be recommended to all hospitals. With it a constant supply of boiling water only is to be had. It is therefore especially useful for night work in hospitals. The gas consumption being small, it is really economical in use. The man in the street will perhaps turn with greater interest to the exhibit of furniture by Messrs. Wolfe and Hollander, whose small table, portable and handy as a tea-tray, is well worthy the attention of hospital authorities. It is exceedingly useful to nurses, and can be recommended to hotels for taking up an early cup of tea. It should be in every home ready not only for emergency but for everyday use.

